# A three‐wave longitudinal study of subcortical–cortical resting‐state connectivity in adolescence: Testing age‐ and puberty‐related changes

**DOI:** 10.1002/hbm.24630

**Published:** 2019-05-17

**Authors:** Anna C. K. van Duijvenvoorde, Bianca Westhoff, Frank de Vos, Lara M. Wierenga, Eveline A. Crone

**Affiliations:** ^1^ Institute of Psychology Leiden University Leiden The Netherlands; ^2^ Leiden Institute for Brain and Cognition Leiden University Leiden The Netherlands

**Keywords:** adolescence, functional connectivity, longitudinal, pubertal development, resting‐state

## Abstract

Adolescence is the transitional period between childhood and adulthood, characterized by substantial changes in reward‐driven behavior. Although reward‐driven behavior is supported by subcortical‐medial prefrontal cortex (PFC) connectivity, the development of these circuits is not well understood. Particularly, while puberty has been hypothesized to accelerate organization and activation of functional neural circuits, the relationship between age, sex, pubertal change, and functional connectivity has hardly been studied. Here, we present an analysis of resting‐state functional connectivity between subcortical structures and the medial PFC, in 661 scans of 273 participants between 8 and 29 years, using a three‐wave longitudinal design. Generalized additive mixed model procedures were used to assess the effects of age, sex, and self‐reported pubertal status on connectivity between subcortical structures (nucleus accumbens, caudate, putamen, hippocampus, and amygdala) and cortical medial structures (dorsal anterior cingulate, ventral anterior cingulate, subcallosal cortex, frontal medial cortex). We observed an age‐related strengthening of subcortico‐subcortical and cortico‐cortical connectivity. Subcortical–cortical connectivity, such as, between the nucleus accumbens—frontal medial cortex, and the caudate—dorsal anterior cingulate cortex, however, weakened across age. Model‐based comparisons revealed that for specific connections pubertal development described developmental change better than chronological age. This was particularly the case for changes in subcortical–cortical connectivity and distinctively for boys and girls. Together, these findings indicate changes in functional network strengthening with pubertal development. These changes in functional connectivity may maximize the neural efficiency of interregional communication and set the stage for further inquiry of biological factors driving adolescent functional connectivity changes.

## 1 INTRODUCTION

Adolescence is a transitional period linking childhood and adulthood, and is accompanied by long‐lasting, largely asynchronous brain changes in both cortical and subcortical brain regions. It is particularly relevant to consider these transformations not only in relation to neural structural or localized activation changes, but also in relation to functional connectivity changes in the adolescent brain (Casey, 2015; Crone & Dahl, 2012; Stevens, 2016). Relative to studies examining structural (Tamnes et al., 2017) and functional (Telzer et al., 2018) brain development, very few studies have examined longitudinal functional connectivity changes. The goal of this study was therefore to analyze within and between subcortical–cortical connectivity in participants ages 8–29 years, using a three‐wave longitudinal design covering 5 years for each individual.

Central to adolescent brain development is a change in the neural motivational circuitry (Doremus‐Fitzwater & Spear, 2016; Ernst, 2014; Telzer, 2016; van Duijvenvoorde, Peters, Braams, & Crone, 2016), which may lead to an increased drive for reward and enhanced affective responses during adolescence, and may create vulnerabilities for developing psychopathology (Paus, Keshavan, & Giedd, 2008). The ventral striatum, and particularly the nucleus accumbens, is considered a key structure for reward processing. This structure is extensively connected to both cortical and subcortical structures supporting motivated behavior (Alexander, Crutcher, & DeLong, 1990; Haber & Knutson, 2010), through looped cortical–subcortical connections. The medial prefrontal cortex (PFC) is densely connected to the ventral striatum and suggested to be a crucial regulator of reward‐directed behavior. In addition, the amygdala and hippocampus are also central regions for respectively affective processing (e.g., Scherf, Smyth, & Delgado, 2013), processing aversive stimuli (Ernst, 2014), and memory (e.g., Davidow, Foerde, Galván, & Shohamy, 2016) in adolescents, and are often coactivated with the medial PFC. Thus, the ventral striatum together with nuclei in the amygdala, parts of the hippocampus, and the medial PFC, form a larger circuitry that modulate responses to salient stimuli and drive reward learning and decision‐making.

This study examined the functional coupling between key reward regions using resting‐state (RS) functional magnetic resonance imaging (fMRI), which provides an important framework for investigating functional systems in the organization of the adolescent developing brain (Ernst, Torrisi, Balderston, Grillon, & Hale, 2015; Uddin, Supekar, & Menon, 2010), considering the minimal experimental demands. Previous RS studies already observed developmental changes in functional connectivity between subcortical regions and prefrontal circuitry. Whereas the functional coupling between the amygdala and medial PFC has been found to increase (Gabard‐Durnam et al., 2014) or show minimal changes (Peters, Peper, Duijvenvoorde, Braams, & Crone, 2016) across adolescence, studies also found a developmental *decrease* in connectivity strength for other subcortical–cortical connections, such as connectivity between the ventral striatum and PFC (Fareri et al., 2015; Padmanabhan, Lynn, Foran, Luna, & O'Hearn, 2013; Porter et al., 2015; van Duijvenvoorde, Achterberg, Braams, Peters, & Crone, 2016). This decrease in functional coupling between subcortical and prefrontal circuitries has been interpreted as a maturation of brain networks, and linked to a developmental decrease in risky behavior and reward valuation (van Duijvenvoorde, Achterberg, et al., 2016) across adolescence, but also to individual differences in risky behavior. For instance, greater functional coupling between the ventral striatum and PFC has been related to an earlier onset of substance use in adolescence (Weissman et al., 2015), and a family history of alcoholism (Cservenka, Casimo, Fair, & Nagel, 2014). However, these studies used different age samples, cross‐sectional designs, and focused on single connections. Therefore, it remains to be determined how functional connectivity changes within and between several subcortical–cortical connections. Moreover, it has often been assumed that heightened subcortical reactivity is related to pubertal onset (Braams, van Duijvenvoorde, Peper, & Crone, 2015; Pfeifer et al., 2011), and pubertal development is suggested to be the maturational process driving developmental changes in *reward* regions, accelerating typical developing trajectories (Blakemore, Burnett, & Dahl, 2010; Crone & Dahl, 2012; Schulz & Sisk, 2016; Vijayakumar, Op de Macks, Shirtcliff, & Pfeifer, 2018). Only a handful of studies tested the influence of pubertal development on subcortical–cortical functional connectivity (Fareri et al., 2015; Peters, Jolles, van Duijvenvoorde, Crone, & Peper, 2015). These studies highlighted that higher pubertal hormone concentrations were linked to a decrease in subcortical–prefrontal connectivity strength as seen across typical adolescent development. However, to date, no study has examined the relative contributions of age and puberty on functional connectivity changes.

In sum, a reorganization of subcortical–cortical circuitry in adolescence is integral to adolescent development. However, few studies have yet examined connectivity in regions of the adolescent reward circuitry in a comprehensive maturational perspective. Here, we related subcortical–cortical circuitry to age and pubertal development in a three‐wave longitudinal sample (8–29 years). Longitudinal accelerated designs consider individual trajectories, thereby allowing for a more accurate estimate of developmental change. RS functional connectivity was examined between a set of subcortical and cortical structures of interest, which included the ventral and dorsal striatum, putamen, hippocampus, amygdala, and all atlas‐based anatomical regions of the medial PFC (subcallosal cortex, ventral medial PFC, anterior cingulate cortex [ACC]). We expected a decoupling between subcortical and medial PFC regions with age, which may be particularly driven by pubertal‐related changes. Given the large sample size and intensive longitudinal measurements, this study also allowed us to examine sex differences and age by sex interactions in RS connectivity change.

## 2 METHODS

### 2.1 Participants

The current study was part of BrainTime, a longitudinal study from Leiden University, Leiden, the Netherlands. Participants were recruited through local schools and advertisements and provided written informed consent for the study at every time point (participant assent and parental consent in case of minors). Participants were screened for MRI contraindications and had no neurological or psychiatric disorders at time point 1 (T1). All anatomical MRI scans were reviewed by a radiologist and no anomalous findings at any of the time points were reported. At each time point, participants received an endowment for participation in a larger scale study. The RS data presented here were collected as the first scan of the BrainTime experimental protocol examining affective and cognitive development via the use of task‐based functional neuroimaging. The study and its procedures were approved by the institutional review board of the Leiden University Medical Center. Cross‐sectional RS analyses have previously been reported in van Duijvenvoorde, Achterberg, et al. (2016) for nucleus accumbens–prefrontal connectivity, and two data waves have previously been reported in Peters, Peper, et al. (2016) for amygdala–PFC connectivity.

At T1, MRI data were collected from 299 participants (*M*_age_ = 13.98 years; *SD*_age_ = 3.68; range = 8.01–25.95 years; 146 males), who were invited for time point 2 (T2) approximately 2 years after T1 (*M*_time‐difference_ = 1.99 years; *SD*_*t*ime‐difference_ = 0.10; range = 1.66–2.47 years). T2 MRI data were collected from 255 participants (32 excluded due to braces, 12 unwilling to participate again). All participants were invited for time point 3 (T3), approximately 2 years after T2 (*M*_time‐difference_ = 2.02 years; *SD*_time‐difference_ = 0.09; range = 1.62–2.35 years). At T3, 243 participants participated in the MRI session (32 excluded due to braces, 24 unwilling to participate again).

Exclusion from further analyses occurred due to a number of reasons. First, participants were excluded when either the RS scan, high‐resolution scan, or T1‐weighted anatomical scan was missing or failed due to technical errors (T1: *n* = 5, T2: *n* = 2; T3: *n* = 4). Second, participants were excluded from all time points if they were diagnosed with a neurological or psychiatric disorder (e.g., depression, Attention‐Deficit Hyperactivity Disorder, Attention‐Deficit Disorder, anxiety disorder) at T2 and/or T3 (*n* = 21). Third, participants were excluded when excessive head motion was detected (T1: *n* = 38; T2: *n* = 23; T3: *n* = 10). Motion exclusion was based on having ≥2 mm translation or more than 2° rotation in any direction, having ≥10 volumes (with more than 0.5 mm movement between two frames (framewise displacement, FD, Power et al., 2014), and/or having ≥10 volumes that are reference RMS outliers (i.e., Root mean square intensity difference of volume *N* to the reference volume, exceeding the threshold of 75th percentile + 1.5 × interquartile range). FD and reference RMS outliers were established using the motion outlier tool implemented in FMRIB Software Library (FSL) version 5.0.4 (http://fsl.fmrib.ox.ac.uk/fsl/fslwiki/, Smith et al., 2004).

The final sample consisted of 661 observations from 273 participants. Specifically, 236 participants at T1 (114 males; *M*_age_ = 14.13, *SD*_age_ = 3.6; range = 8–25 years), 211 participants at T2 (101 males; *M*_age_ = 16.2, *SD*_age_ = 3.48; range = 10–26 years), and 214 participants at T3 (100 males; *M*_age_ = 17.93, *SD*_age_ = 3.52; range = 12–28 years). Table 1 also summarizes the number of participants in T1, T2, and T3, and the main subject characteristics.

**Table 1:** Subject characteristics

	Time point	Males	Females
Age (min–max)		8–28 years	8–26 years
Total number of scans		316	345
Number participants contributing one data point		25	16
Number participants contributing two data points		33	46
Number participants contributing three data points		75	79
PDS mean (*SD*)	1	2.15 (0.78)	2.45 (0.97)
	2	2.5 (0.75)	2.96 (0.76)
	3	2.85 (0.75)	3.22 (0.6)
FD mean (*SD*)	1	0.148 (0.05)	0.148 (0.05)
	2	0.157 (0.04)	0.164 (0.04)
	3	0.097 (0.03)	0.097 (0.03)
FD FIX‐denoised mean (*SD*)	1	0.037 (0.01)	0.035 (0.01)
	2	0.033 (0.01)	0.033 (0.01)
	3	0.03 (0.01)	0.029 (0.01)

Abbreviations: FD = framewise displacement; PDS = Pubertal Development Scale.

Intelligence quotient (IQ) was estimated at T1 using the subsets “similarities” and “block design” and at T2 using the subsets “Vocabulary” and “Picture Completion” of the Wechsler Intelligence Scale for Adults (WAIS‐III) or the Wechsler Intelligence Scale for Children, third edition (WISC‐III; Wechsler, 1974). All estimated IQ scores were in the normal range on T1 (*M*_IQ_ = 109.9, *SD*_IQ_ = 10.7, range = 80–143) and T2 (*M*_IQ_ = 108.4, *SD*_IQ_ = 10.3, range = 80–148) and were not significantly related to age at either time point (all *p*s > .4) for included subjects.

### 2.2 Pubertal stage

Stage of physical pubertal maturation was assessed at each time point with the Pubertal Development Scale (PDS) for participants under 18 years of age (Petersen et al. 1988). This self‐report questionnaire contains questions concerning secondary sexual characteristics. The participants were instructed to indicate their developmental stage on each of these physical characteristics on a 4‐point scale: ranging from (a) has not started to develop, (b) shows first signs of development, (c) shows clear development to (d) has finished developing. The average score on all items was used for further analysis. Data on PDS scores were included for 405 data points (T1: *n* = 185; T2: *n* = 119; T3: *n* = 101). This questionnaire was only administered to participants up to 18 years of age (>18 years T1: *n* = 28; T2: *n* = 59; T3: *n* = 93), because it was assumed that all participants completed pubertal development by 18 years. Other missing data occurred because of administration or technical errors (missing T1: *n* = 23; T2: *n* = 23; T3: *n* = 20). Longitudinal data of the PDS have been reported on this sample for two waves in Braams et al. (2015) and three waves in Peper, Braams, Blankenstein, Bos, and Crone (2018), and Wierenga et al. (2018). Pubertal development and age were highly correlated at each time point: T1: *r* (185) = .785, *p* < .001; T2: *r* (129) = .739, *p* < .001; T3: *r* (101) = .638, *p* < .001.

### 2.3 MRI data acquisition

Neuroimaging was conducted using a 3.0 T Philips Achieva MRI scanner with a standard whole‐head coil. The same scanner and settings were used for all participants and at all three time points. The RS scans were acquired as the first scan of a fixed‐order imaging protocol, with T2*‐weighted echo‐planar imaging (EPI) (140 volumes; 38 slices; sequential acquisition; time repetition (TR) = 2,200 ms, time echo (TE) = 30 ms; flip angle = 80°; field of view (FOV) = 220 × 220 × 114.67 mm^3^; slice thickness = 2.75 mm). Two additional dummy scans preceded the scan to allow for equilibration of T1 saturation effects. Participants were instructed to lie still with their eyes closed, without falling asleep.

For registration purposes, we additionally obtained a high‐resolution T2*‐weighted gradient EPI scan (84 slices; TR = 2,200 ms; TE = 30 ms; flip angle = 80°; FOV = 220 × 220 × 168 mm^3^; in‐plane resolution = 1.96 × 1.96; slice thickness = 2 mm), and a T1‐weighted anatomical scan (140 slices; TR = 9.76 ms; TE = 4.59 ms; flip angle = 8°; FOV = 224 × 177.33 × 168 mm^3^; in‐plane resolution = 0.875 × 0.875 mm; slice thickness = 2 mm), at the end of a fixed imaging protocol which included functional tasks.

### 2.4 fMRI data preprocessing

The RS functional data were preprocessed using FEAT (fMRI Expert Analysis Tool; v6.00), part of FSL (Smith et al., 2004). Preprocessing of the RS data included motion correction (MCFLIRT; Jenkinson, Bannister, Brady, & Smith, 2002), slice timing correction (regular down), brain extraction (BET), spatial smoothing with a 5 mm full‐width‐at‐half‐maximum Gaussian kernel, and high‐pass temporal filtering with a cutoff point of 100 s. The high‐resolution EPI images and T1‐weighted anatomical images were brain‐extracted (BET). Next, the RS fMRI scans of an individual were registered to the corresponding high‐resolution EPI image (6 DOF), which in turn were registered to the T1‐weighted anatomical image using the integrated version of boundary‐based registration to improve the accuracy of functional‐to‐structural space registration. Finally, the images were registered to standard MNI‐152 space using FNIRT (FMRIB's Nonlinear Imaging Registration Tool; 12 DOF, warp resolution 10 mm).

### 2.5 Motion correction

Head motion is undesirable in all fMRI studies (e.g., Friston, Williams, Howard, Frackowiak, & Turner, 1996), and especially so for RS studies, as head motion may overestimate short‐distance correlations and underestimate long‐distance correlations (Power, Barnes, Snyder, Schlaggar, & Petersen, 2012; van Dijk, Sabuncu, & Buckner, 2012). Developmental samples are particularly susceptible for this confound, given that head motion is highly related to subject age (Satterthwaite et al., 2013, 2017).

To minimize motion, subjects were trained with a mock‐scanning procedure, were reminded several times during the session not to move during scanning, and head motion was restricted using foam padding. We applied a strict exclusion criterion (see Section 2.1) based on absolute motion, an FD cutoff of 0.5 mm on ≥10 volumes, and/or reference RMS outliers on ≥10 volumes. Although the mean FD was relatively low on each time point (see Table 1), the mean FD correlated significantly with age on T1 (*r*_(236)_ = −.142, *p* = .029 and T3 (*r*_(213)_ = −.15, *p* = .025). To minimize these potential influences of head motion, we denoised the preprocessed RS data of the included participants with FIX (FMRIB's ICA‐based Xnoiseifier, version 1.06) using the included standard training data set (threshold 15) (Griffanti et al., 2014; Salimi‐Khorshidi et al., 2014). FIX classifies ICA components and automatically removes the noise components (e.g., result of motion) from the RS time series. This resulted in a clear lowering of the mean FD (see Table 1). Note that for these cleaned time series, all included participants adhered even to a more stringent FD cutoff of 0.2 mm on ≥2 volumes.

### 2.6 Nuisance signal regression

In addition to motion artifacts, signals from white matter (WM) and cerebrospinal fluid (CSF) can be confounding effects that result in overestimated RS connectivity strength. These signals primarily reflect noise from non‐neural origin (e.g., scanner instabilities, physiological effects) and are largely independent from Blood Oxygenation Level‐Dependent (BOLD) signal fluctuations in gray matter (Windischberger et al., 2002). Global signal was also removed from the time series to reduce influence of artifacts caused by physiological processes (i.e., cardiac and respiratory fluctuations), vigilance level (Liu, Nalci, & Falahpour, 2017), and scanner drifts (Fox & Raichle, 2007).

WM and CSF masks were obtained using FAST (FMRIB's Automated Segmentation Tool), which segments the T1‐weighted anatomical scan into different tissue types (WM, CSF, and gray matter). These maps were then FLIRT‐based transformed into functional subject space and eroded by one voxel (3 × 3 × 3 mm) to minimize potential partial volume effects. Global signal time series were calculated in native space as the average signal across all nonzero voxels in the brain. WM, CSF, and global signal time series were used as temporal covariates and removed from the RS time series of each ROI at the individual participant level through linear regression in MATLAB. We then calculated Pearson correlations between all ROI time series and transformed them into *Z*‐values using the Fisher *Z*‐transformation in MATLAB.

### 2.7 Regions of interest

**Figure 1:**
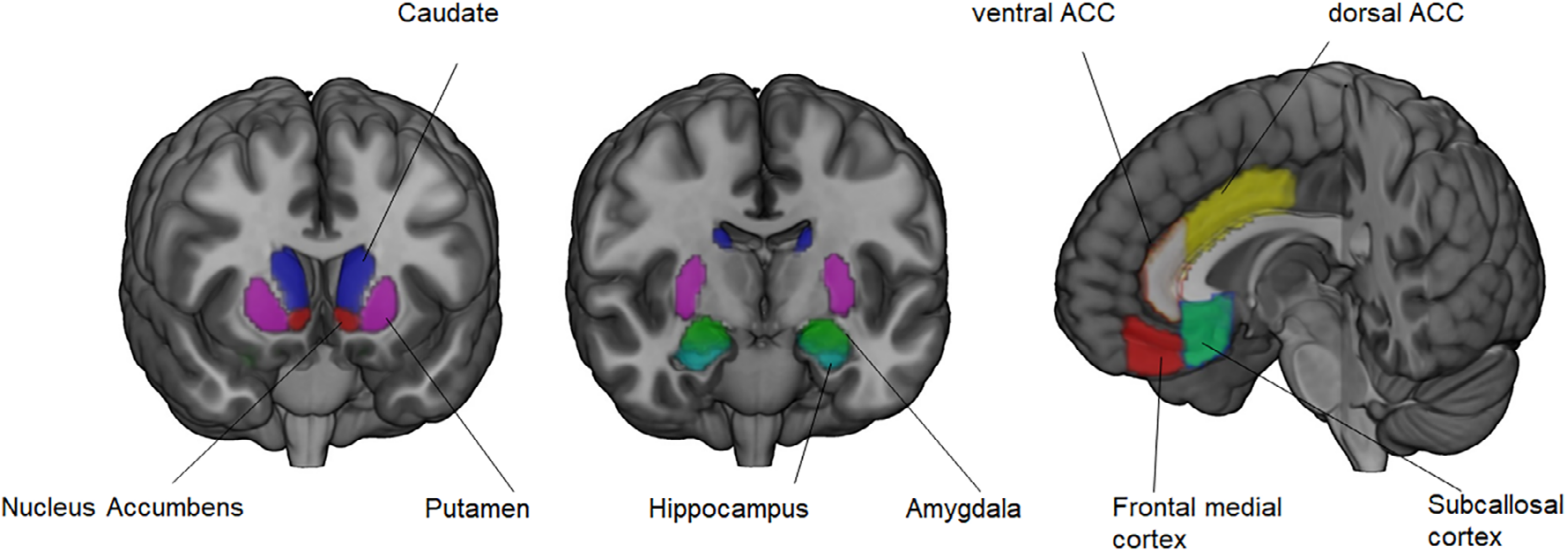
The four cortical midline structures and five subcortical regions of interest [Color figure can be viewed at http://wileyonlinelibrary.com]

Regions of interest (ROIs) were selected from the Harvard–Oxford probabilistic anatomical brain atlas (subcortical and cortical) in FSL, with a thresholded probability of ≥0.5. Although based on anatomical parcellation, the Harvard–Oxford atlas is an often used and well‐known atlas in functional brain analyses. Given our focus on age‐related change in connectivity between frontal midline and subcortical structures, we included four cortical midline structures in this atlas (subcallosal cortex, frontal medial cortex, and cingulate gyrus anterior division) that spanned a ventral to dorsal cortical midline. Considering the extent and functional specificity of the ACC, this anatomical structure was divided in a more posterior‐dorsal and a more anterior‐ventral part with a cutoff of *y* = 30 based on Bush, Luu, and Posner (2000) (see for a similar segregation Achterberg et al., 2018). We included five bilateral subcortical ROIs: nucleus accumbens, caudate, putamen, amygdala, and hippocampus. For each subcortical region, bilateral masks were combined into one ROI for further analyses. ROIs are visualized in Figure 1. For each participant, the ROIs were transformed to subject space and the mean individual RS time series were extracted from each ROI separately.

### 2.8 Experimental design and statistical analysis

Age‐related change in longitudinal data sets is often assessed with polynomial growth models including age as linear, quadratic, or cubic regressor, while controlling for the repeated nature of the data. However, a limitation of these models is that they assume age‐related changes follow this restricted set of growth models. Additionally, these models may not be optimal to compare groups that show different developmental trajectories (Vijayakumar, Op de Macks, et al., 2018). Thus, we used a distinct class of models called generalized additive mixed models (GAMMs) to characterize age‐ and sex effects, pubertal‐related effects, and behavioral effects on RS functional connectivity. All models were run using the mgcv package (Wood, 2011) in R (R Core Team, 2017; https://www.r-project.org/). GAMM is similar to a generalized linear mixed model where predictors can be replaced by smooth functions of themselves, offering efficient and flexible estimation of nonlinear effects. Smooth splines can capture important nonlinear changes that are easily missed with polynomials, prevent biased fits at the extreme ranges of the data, while controlling Type 1 error rate in AIC/ Bayesian Information Criterion (BIC) values (Wood, 2017) and *p*‐values (Wood, 2013). Moreover, GAMM models are well suited for our developmental sample and accelerated longitudinal design, as this model accounts for within‐subject dependence and differences in developmental time points at which participants entered the study (Alexander‐Bloch et al., 2014; Harezlak, Ryan, Giedd, & Lange, 2005; Wierenga et al., 2018).

Our research questions followed the following model‐fit procedures. First, to assess age‐ and sex‐related change in functional connectivity we used a model‐building procedure assessing (a) the developmental age‐related pattern over the whole group; (b) a main effect of sex; and (c) differences in developmental trajectories between sexes. These models were compared to test which model provided the best fit for each connection. In short, first a simple age model of formula (1) was fit, where *s* () represents a penalized smoothing spline. A fixed overall intercept and a random intercept per participant were included in all models. The latter accounts for the repeated nature of the data. All models included a residual error term.

(1) $$\text{GAMM}\;(\text{Connection}∼s\left(\mathrm{Age}\right),\;\text{random}=\text{list}\;\left(\text{Subject}=∼1\right)$$

Expanded models were fit to include a fixed main effect of Sex (Equation 2) *,* and a Sex by Age interaction (Equation 3)

(2) $$\text{GAMM}\;(\text{Connection}∼\mathrm{Sex}+s\left(\mathrm{Age}\right),\;\text{random}=\text{list}\;\left(\text{Subject}=∼1\right)$$

(3) $$\text{GAMM}\;(\text{Connection}∼\mathrm{Sex}+s\left(\mathrm{Age}\right)+s\left(\mathrm{Age}*\mathrm{Sex}\right),\;\text{random}=\text{list}\;\left(\text{Subject}=∼1\right)$$

The dimension used to represent the smooth terms *k* was limited to a maximum of four in all models. *k* should be set large enough to have enough degrees of freedom to represent the underlying “true” change, but small enough to maintain reasonable computational efficiency. Based on previous studies using structural MRI measures in a partly overlapping data set (see Wierenga et al., 2018), we used a *k* of 4 as an optimal threshold. Models were compared using the BIC. The model with the lowest BIC value (1, 2, 3) was selected as the best fitting model.

A second aim was to assess puberty‐related effects on RS functional connectivity, and most interestingly, whether this was also a better predictor of RS connectivity than chronological age. First, a simple GAMM model to visualize effect of sex and age on PDS development was tested with the following model (4).

(4) $$\text{GAMM}\;(\mathrm{PDS}∼\mathrm{Sex}+s\left(\mathrm{Age}\right),\text{random}=\text{list}\;\left(\text{Subject}=∼1\right)$$

**Figure 2:**
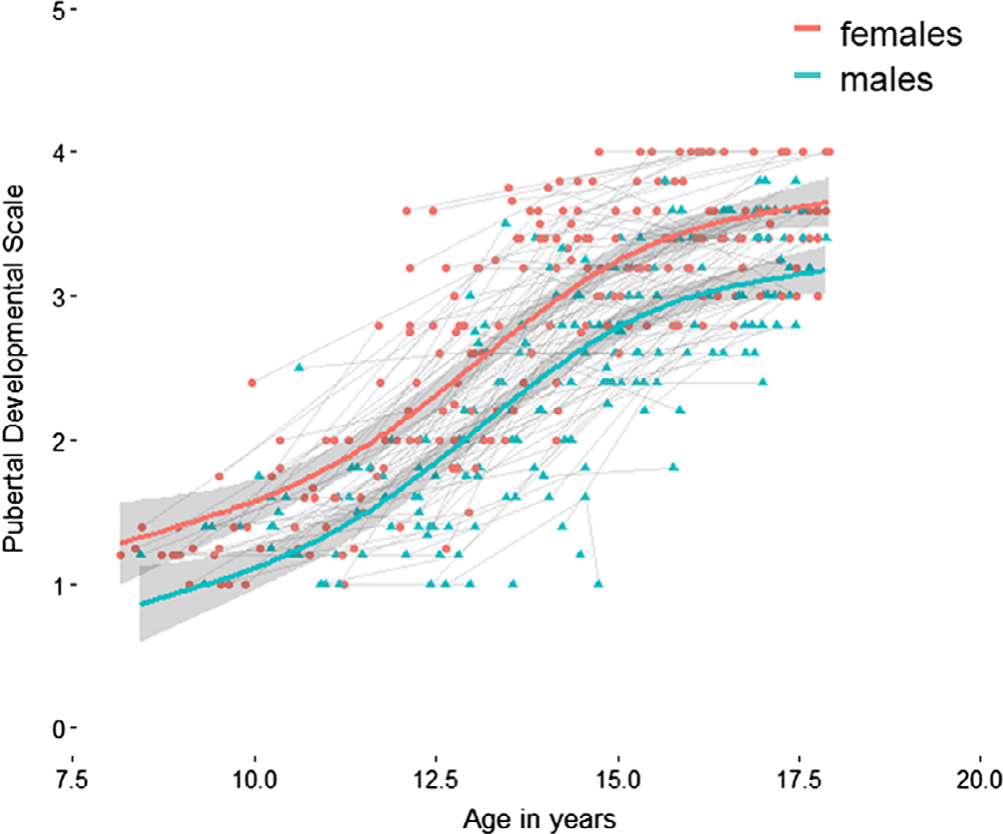
Pubertal Development Scale (PDS) score across age in years. Plot indicates fitted lines of PDS from a generalized additive mixed model for males (blue) and females (red) separately on top of the raw longitudinal data [Color figure can be viewed at http://wileyonlinelibrary.com]

Results showed that PDS score was described by a main effect of sex and spline effect of age. That is, pubertal development manifested differently for boys and girls and increased with age (see Figure 2).

To assess effects of puberty on RS connectivity, models were assessed separately for boys and girls, given that puberty had different timings in males and females (see Wierenga et al., 2018 for an example on structural brain development and pubertal development). We first examined significant effects of PDS by creating a model including a smooth PDS term (Equation (5)).

(5) $$\text{GAMM}\;(\text{Connection}∼s\left(\mathrm{PDS}\right),\text{random}=\text{list}\;\left(\text{Subject}=∼1\right)$$

Testing the robustness of these PDS findings, we compared the BIC of this simple PDS‐only model to a developmental model including chronological age (Equation (6)). Second, for all connections in which PDS was a significant predictor, we also tested whether results of PDS on functional connectivity remained significant after including age as a covariate in the developmental model. Due to the high collinearity between age and PDS, we opted for age at baseline (Equation (7)).

(6) $$\text{GAMM}\;(\text{Connection}∼s\left(\mathrm{Age}\right),\text{random}=\text{list}\;\left(\text{Subject}=∼1\right)$$

(7) $$\text{GAMM}\;(\text{Connection}∼s\left(\mathrm{PDS}\right)+s\;\left({\mathrm{Age}}_{\text{baseline}}\right),\text{random}=\text{list}\;\left(\text{Subject}=∼1\right)$$

The *p*‐values of fixed effects in all best‐fitting models were corrected for multiple comparisons with a Bonferroni–Holm correction and evaluated at *p* < .05.

As a measure of homogeneity of the data, we determined the intraclass correlations (ICCs) for each RS connection. ICCs were computed by estimating a null model (Equation (8)8) with maximum likelihood across all data points and dividing the variance in intercept by the sum of the variance in intercept and residual variance. ICCs are listed in Table 2 and range from very poor (<.1) to poor (<.41) (Cicchetti, 1994).

(8) $$\mathrm{lme}\;(\text{Connection}∼\text{random}=\text{list}\;\left(\text{Subject}=∼1\right)$$

**Table 2:** ICC for each RS functional connection

Connection	ICC
Amygdala–frontal medial cortex	.083
Caudate–nucleus accumbens	.092
Amygdala–caudate	.113
Hippocampus–putamen	.129
Putamen–nucleus accumbens	.132
Hippocampus–nucleus accumbens	.137
Putamen–frontal medial cortex	.139
Caudate–frontal medial cortex	.144
Caudate–ventral ACC	.147
Subcallosal cortex–dorsal ACC	.156
Putamen–dubcallosal cortex	.167
Putamen–ventral ACC	.168
Hippocampus–dorsal ACC	.169
Hippocampus–caudate	.169
Hippocampus–amygdala	.179
Amygdala–putamen	.182
Amygdala–subcallosal cortex	.188
Frontal medial cortex–dorsal ACC	.2
Amygdala–ventral ACC	.206
Hippocampus–ventral ACC	.211
Nucleus accumbens–frontal medial cortex	.213
Hippocampus–subcallosal cortex	.223
Caudate–subcallosal cortex	.226
Amygdala–nucleus accumbens	.226
Caudate–dorsal ACC	.239
Putamen–caudate	.239
Amygdala–dorsal ACC	.25
Nucleus accumbens–ventral ACC	.255
Hippocampus–frontal medial cortex	.267
Putamen–dorsal ACC	.284
Frontal medial cortex–subcallosal cortex	.298
Nucleus accumbens–dorsal ACC	.298
Nucleus accumbens–subcallosal cortex	.313
Ventral ACC–dorsal ACC	.327
Frontal medial cortex–ventral ACC	.327
Subcallosal cortex–ventral ACC	.336

Abbreviations: ACC = anterior cingulate cortex; ICC = intraclass correlations; RS = resting‐state.

## 3 RESULTS

### 3.1 Average RS functional connectivity

**Figure 3:**
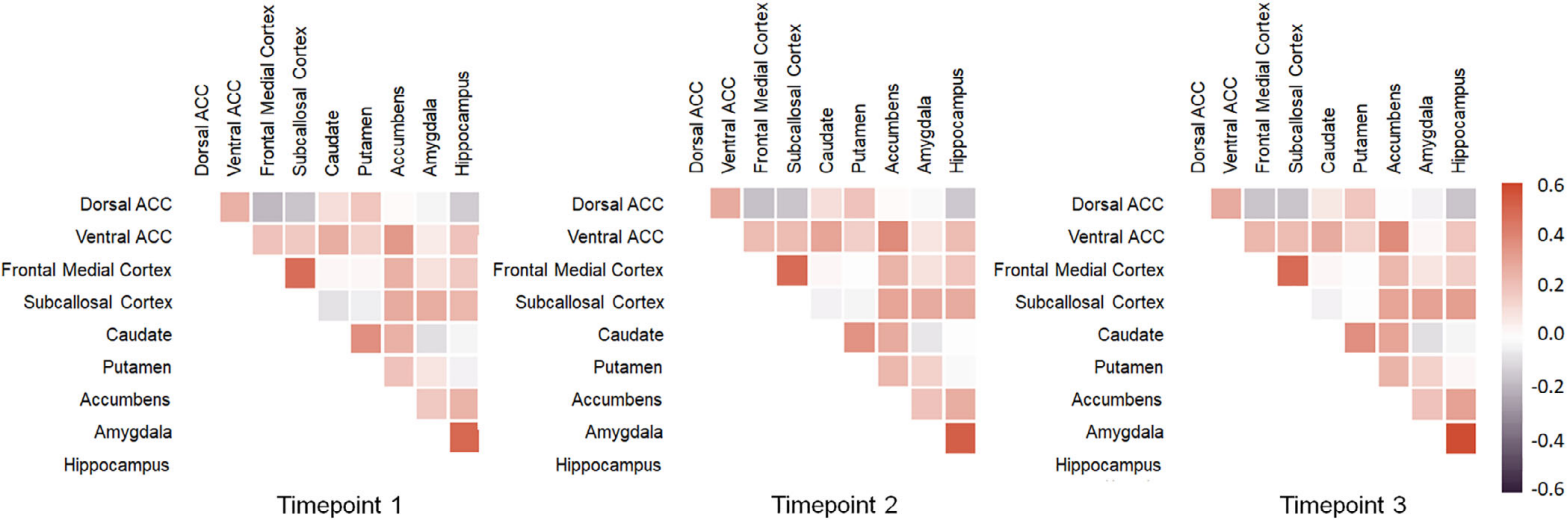
Average full‐correlation matrices (uncorrected Pearson's *r*) between all regions of interest for time point 1, time point 2, and time point 3 [Color figure can be viewed at http://wileyonlinelibrary.com]

Figure 3 displays the average RS‐connectivity strength (Pearson's *r*) for each connection of interest per time point. Most regions were positively functionally connected, with the strongest positive connections between the amygdala–hippocampus and subcallosal cortex–frontal medial cortex. Negative connectivity was observed between dorsal and ventral cortical regions, such as the dorsal ACC–subcallosal cortex, and dorsal ACC–frontal medial cortex.

### 3.2 Age‐related change in RS functional connectivity

**Figure 4:**
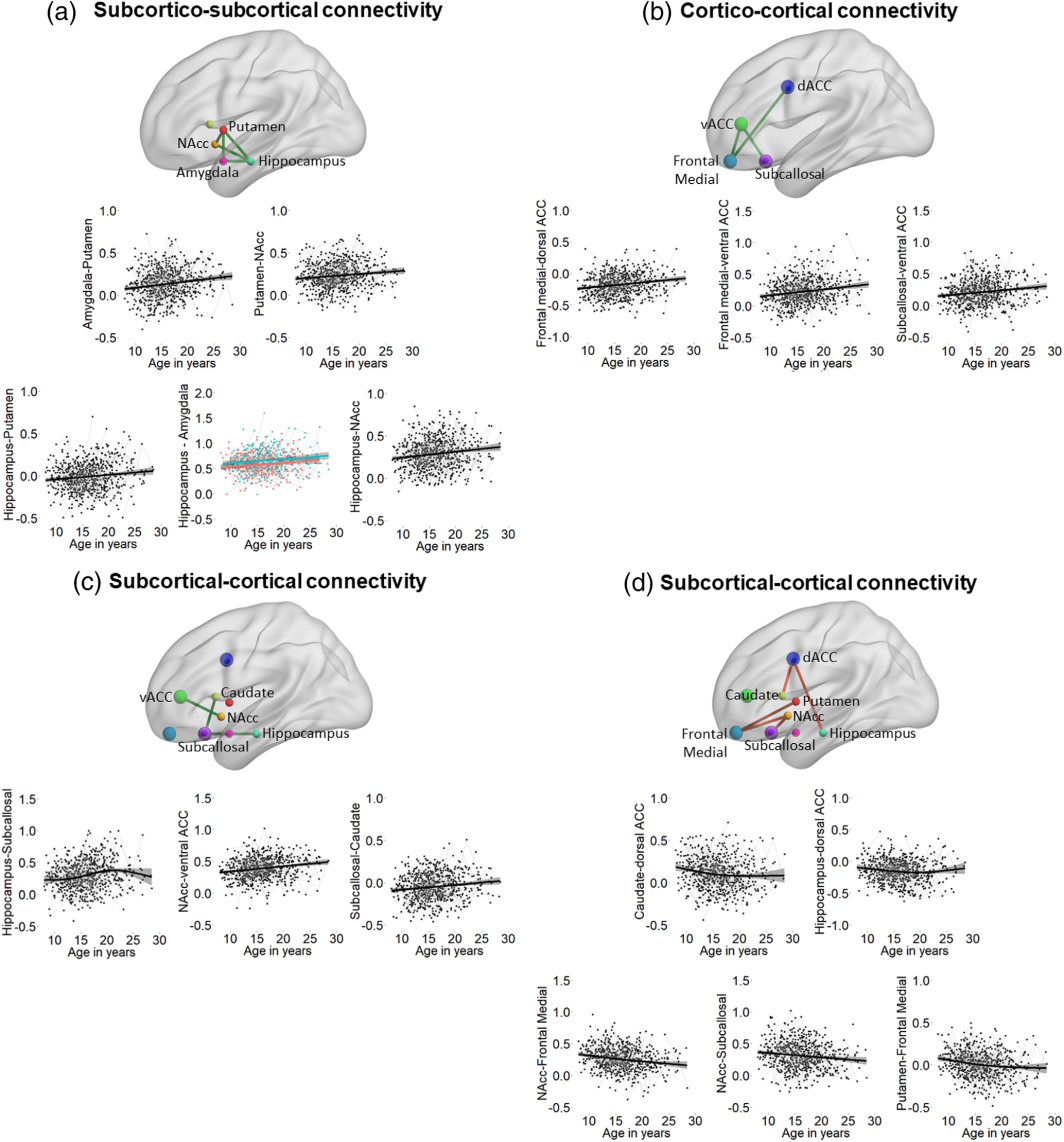
Spaghetti plots indicating a significant fitted line of age on top of the raw longitudinal data for (a) cortico‐cortical connections (b) and subcortical–cortical connections (c,d). Location of region of interest (ROI) is indicated schematically as dots, visualized with the BrainNet viewer (Xia, Wang, & He, 2013, http://www.nitrc.org/projects/bnv/). Green lines between ROIs indicate age‐related increases, and red lines indicate age‐related decreases. If there was a sex difference in functional connectivity development, males are plotted in blue and females in red in spaghetti plots (only the case for hippocampus–amygdala connectivity) [Color figure can be viewed at http://wileyonlinelibrary.com]

We assessed age‐related changes in RS connectivity using GAMM (see Table 3). After correction for multiple comparisons (Bonferroni–Holm), significant positive effects of age were observed for subcortical–subcortical connectivity between the amygdala, putamen, and hippocampus, between the putamen, nucleus accumbens, and hippocampus, and between the hippocampus and nucleus accumbens (Figure 4a), all showing a strengthening of functional connectivity with age. Cortico‐cortical connections also showed a significant strengthening of connectivity with age between the frontal medial cortex–ventral ACC, frontal medial cortex–dorsal ACC, and between the subcallosal cortex–ventral ACC (Figure 4b). Age‐related strengthening of connectivity was observed in subcortical–cortical connectivity, between the hippocampus–subcallosal cortex, caudate–subcallosal cortex, and nucleus accumbens–ventral ACC (Figure 4c). Finally, age‐related decreases were observed only for subcortical–cortical connectivity (Figure 4d), particularly between the caudate–dorsal ACC, hippocampus–dorsal ACC, nucleus accumbens–frontal medial cortex, nucleus accumbens–subcallosal cortex, and putamen–frontal medial cortex. Visualization of significant age splines showed a relatively linear developmental pattern for all connections, with a few subcortical–cortical connections leveling off in adolescence or young adulthood (see Figure 4).

**Table 3:** Generalized additive mixed models examining effects of age and sex. For each connection, model fits are shown for (1) Age‐only models (2) Age + sex modes, and (3) Age x sex models. Corrected P‐values are shown for age (and sex) effects

	BIC values			Model results			
Measure	Model fits				Age spline	Age spline + sex	
	Age	Age + sex	Age × sex	Best model	*p*‐Value	*p*‐Value sex	*p*‐Value age
Ventral ACC–dorsal ACC	−241.16	−236.98	−224.01		.9092		
Subcallosal ACC–dorsal ACC	−390.54	−384.84	−371.99		.4244		
Subcallosal ACC–ventral ACC	−324.90	−318.42	−306.14	Age only	.0019*		
Frontal medial–dorsal ACC	−429.80	−423.31	−411.65	Age only	.0002*		
Frontal medial–ventral ACC	−282.78	−281.30	−273.82	Age only	.0002*		
Frontal medial–subcallosal ACC	−45.86	−40.07	−27.14		.2168		
Accumbens–dorsal ACC	−489.09	−482.92	−470.38		.5770		
Accumbens–ventral ACC	−448.41	−444.37	−432.70	Age only	.0002*		
Accumbens–subcallosal ACC	−198.25	−192.23	−179.46	Age only	.0112*		
Accumbens–frontal medial	−398.37	−396.72	−383.97	Age only	.0002*		
Caudate–dorsal ACC	−390.03	−384.19	−373.74	Age only	.0118*		
Caudate–ventral ACC	−439.57	−435.67	−422.79		.7301		
Caudate–subcallosal ACC	−553.08	−547.02	−534.10	Age only	.0046*		
Caudate–frontal medial	−591.08	−589.00	−576.06		.4244		
Caudate–accumbens	−525.75	−519.33	−507.80		.0989		
Putamen–dorsal ACC	−426.98	−422.88	−409.89		.7122		
Putamen–ventral ACC	−545.97	−539.51	−529.75		.6996		
Putamen–subcallosal ACC	−516.83	−510.50	−498.20		.2561		
Putamen–frontal medial	−587.49	−586.12	−573.15	Age only	.0103*		
Putamen–accumbens	−597.48	−591.01	−578.17	Age only	.0103*		
Putamen–caudate	−418.32	−413.57	−406.01		.7423		
Amygdala–dorsal ACC	−329.89	−323.56	−310.75		.2827		
Amygdala–ventral ACC	−283.46	−278.10	−265.41		.4734		
Amygdala–subcallosal ACC	−24.79	−18.82	−7.90		.6911		
Amygdala–frontal medial	−407.55	−402.03	−392.56		.0917		
Amygdala–Accumbens	−392.82	−386.61	−374.90		.1170		
Amygdala–caudate	−462.45	−458.18	−445.29		.2561		
Amygdala–putamen	−432.42	−426.03	−413.36	Age only	.0005*		
Hippocampus–dorsal ACC	−391.67	−385.67	−372.90	Age only	.0409*		
Hippocampus–ventral ACC	−241.72	−251.99	−239.74	Age + sex		.0013*	.8777
Hippocampus–subcallosal ACC	−126.37	−120.27	−107.28		.0001		
Hippocampus–frontal medial	−317.67	−315.85	−303.33		.6117		
Hippocampus–accumbens	−414.21	−407.71	−395.11	Age only	.0018*		
Hippocampus–caudate	−534.12	−527.71	−516.70		.5351		
Hippocampus–putamen	−454.18	−452.57	−439.67	Age only	.0097*		
Hippocampus–amygdala	−145.66	−153.46	−142.16	Age + sex		.0022*	.0028*

Abbreviations: ACC = anterior cingulate cortex; BIC = Bayesian Information Criterion.

*Note*. For all models, corrected *p*‐values are reported (Bonferroni–Holm). *Corrected *p*‐value <.05.

For two connections, the best‐fitting model included a main effect of sex. That is, functional connectivity between hippocampus–amygdala increased with age and was greater for males than females (see Figure 4 plotted in red/blue, and Table 3). Functional connectivity between the hippocampus–ventral ACC was not dependent on age, but was greater for females than males. None of the models showed a best fit for model (3) including age by sex interaction terms.

### 3.3 Pubertal development changes in RS functional connectivity

To assess pubertal developmental changes in RS functional connectivity, a set of GAMMs were run, only including participants between ages 8 and 18‐years old, during which PDS changes are most pronounced. Given the difference in pubertal timing between sex, all models were run separately for boys and girls (see for a similar approach Wierenga et al., 2018).

**Figure 5:**
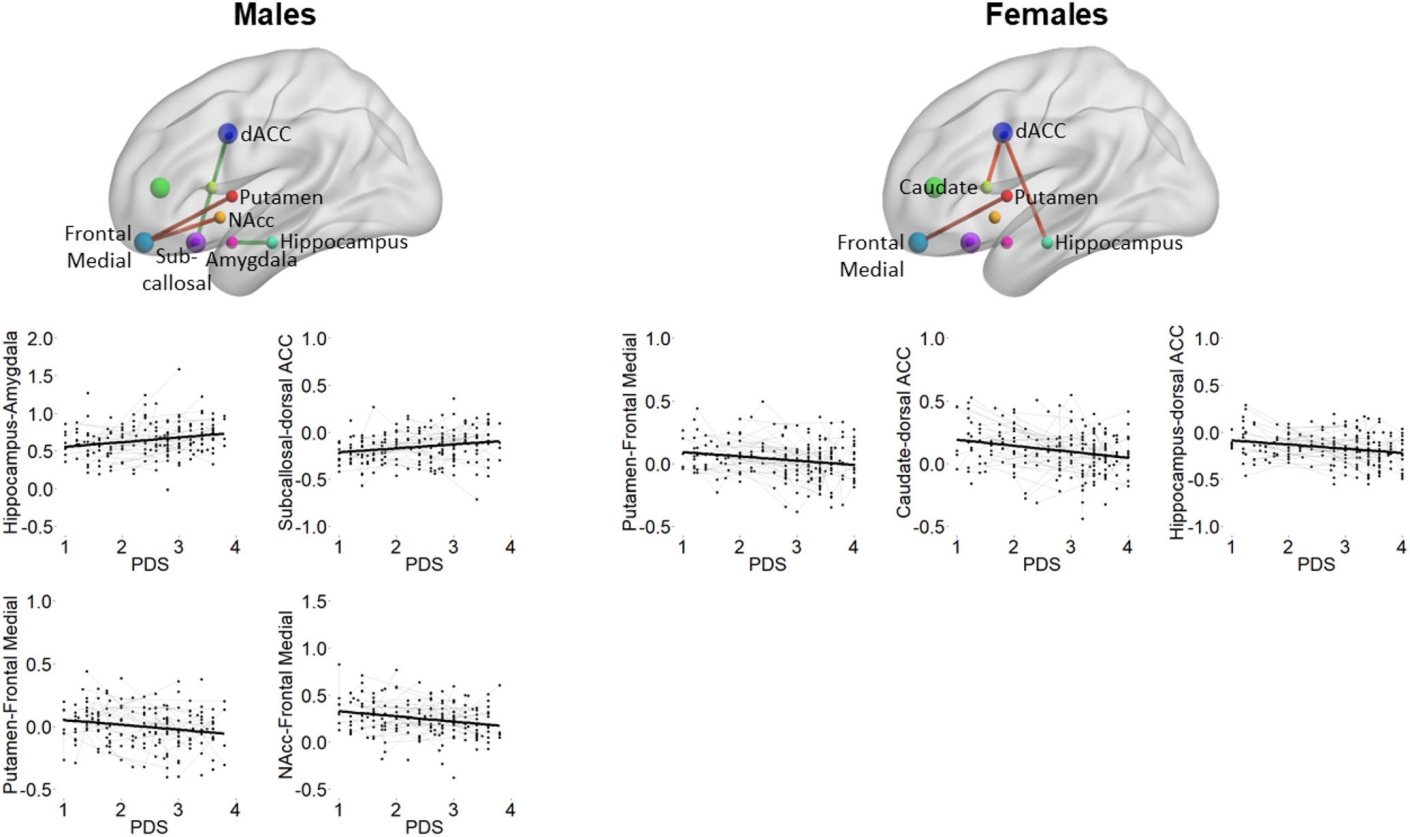
Spaghetti plots indicating a significant fitted line of pubertal development on top of the raw longitudinal data for males (left panel) and females (right panel). Location of region of interest (ROI) is indicated schematically as dots, visualized with the BrainNet viewer (Xia et al., 2013, http://www.nitrc.org/projects/bnv/). Green lines between ROIs indicate Pubertal Development Scale (PDS)‐related increases, and red lines indicate PDS‐related decreases [Color figure can be viewed at http://wileyonlinelibrary.com]

For boys, subcortical–cortical connectivity between the nucleus accumbens–frontal medial cortex and putamen–frontal medial cortex related negatively to PDS scores, with increasing PDS significantly decreasing RS connectivity (similarly to what was observed for age) (see Figure 5; Table 4). Additionally, connectivity between the hippocampus‐amygdala (similar to age effects) and subcallosal cortex–dorsal ACC (not observed for age) was significantly positively related to PDS score, with increasing PDS being associated with increased RS connectivity. The nucleus accumbens‐frontal medial cortex and subcallosal cortex–dorsal ACC connectivity showed a better fit for a PDS‐only than an age‐only model when comparing BICs. When including age at baseline and PDS in the same model, only the hippocampus–amygdala connectivity showed a significant effect of PDS on RS connectivity over and above age at baseline (*p* = .002).

**Table 4:** Generalized additive mixed models examining effects of PDS and age for males. For each connection, model fits are shown for (1) PDS‐only models (2) Age‐only models. Corrected P‐values are shown for effects of PDS

Measure	BIC values		
	Model fits		PDS spline
	PDS	Age	*p*‐Value PDS
Ventral ACC–dorsal ACC	−80.31	−81.43	.6712
Subcallosal ACC–dorsal ACC	−127.92	−125.21	.0433^*^
Subcallosal ACC–ventral ACC	−96.67	−98.09	.0937
Frontal medial–dorsal ACC	−133.59	−137.10	.3167
Frontal medial–ventral ACC	−63.57	−65.79	.1192
Frontal medial–subcallosal ACC	−8.28	−6.16	.1379
Accumbens–dorsal ACC	−161.47	−160.61	.5224
Accumbens–ventral ACC	−112.96	−114.14	.1192
Accumbens–subcallosal ACC	−17.13	−17.14	.6712
Accumbens–frontal medial	−96.22	−92.55	.0217^*^
Caudate–dorsal ACC	−129.58	−125.70	.1192
Caudate–ventral ACC	−96.53	−96.26	.7379
Caudate–subcallosal ACC	−157.21	−158.51	.6712
Caudate–frontal medial	−178.24	−180.44	.5857
Caudate–accumbens	−131.50	−128.59	.0930
Putamen–dorsal ACC	−123.09	−123.66	.9538
Putamen–ventral ACC	−163.21	−163.17	.9384
Putamen–subcallosal ACC	−121.57	−121.63	.3167
Putamen–frontal medial	−158.52	−161.58	.0433^*^
Putamen–accumbens	−143.26	−140.63	.0706
Putamen–caudate	−83.29	−86.45	.7086
Amygdala–dorsal ACC	−59.59	−60.10	.9538
Amygdala–ventral ACC	−70.56	−70.75	.9538
Amygdala–subcallosal ACC	16.22	16.43	.8092
Amygdala–frontal medial	−82.04	−81.08	.3371
Amygdala–accumbens	−83.92	−88.01	.5224
Amygdala–caudate	−143.84	−145.87	.2234
Amygdala–putamen	−93.03	−95.65	.0689
Hippocampus–dorsal ACC	−111.32	−111.81	.9562
Hippocampus–ventral ACC	−78.78	−78.63	.7379
Hippocampus–subcallosal ACC	−26.43	−27.47	.3234
Hippocampus–frontal medial	−96.07	−96.59	.8092
Hippocampus–accumbens	−107.35	−111.65	.8092
Hippocampus–caudate	−137.40	−137.44	.8637
Hippocampus–putamen	−124.95	−124.33	.6053
Hippocampus–amygdala	−30.06	−35.28	.0217^*^

Abbreviations: ACC = anterior cingulate cortex; BIC = Bayesian Information Criterion; PDS = Pubertal Development Scale.

*Note*. Multiple comparison corrected *p*‐values are reported (Bonferroni–Holm). *Corrected *p*‐value <.05.

For girls, subcortical–cortical connectivity between the hippocampus–dorsal ACC (also observed for age), caudate–dorsal ACC (also observed for age), and putamen–frontal medial cortex (also observed for age) was significantly negatively related to PDS scores, with increasing PDS related to decreased RS connectivity (see Figure 5; Table 5). All three connections showed a better fit for a PDS‐only than an age‐only model when comparing BICs. Finally, when including PDS and age at baseline in the same model for these three connections, only the hippocampus–dorsal ACC (*p* = .006) and caudate–dorsal ACC (*p* = .02) showed a significant effect of PDS over and above age at baseline.

**Table 5:** Generalized additive mixed models examining effects of PDS and age for females. For each connection, model fits are shown for (1) PDS‐only models (2) Age‐only models. Corrected P‐values are shown for effects of PDS

Measure	BIC values		
	Model fits		PDS spline
	PDS	Age	*p*‐Value PDS
Ventral ACC–dorsal ACC	−87.27	−87.92	.5354
Subcallosal ACC–dorsal ACC	−120.66	−120.62	.8908
Subcallosal ACC–ventral ACC	−84.29	−93.63	.4784
Frontal medial–dorsal ACC	−106.47	−106.50	.4399
Frontal medial–ventral ACC	−107.93	−108.84	.3231
Frontal medial–subcallosal ACC	6.32	7.92	.3231
Accumbens–dorsal ACC	−160.17	−158.97	.4614
Accumbens–ventral ACC	−116.33	−121.94	.3231
Accumbens–subcallosal ACC	−50.83	−49.53	.4442
Accumbens–frontal medial	−146.85	−148.61	.3231
Caudate–dorsal ACC	−135.04	−131.40	.0092^*^
Caudate–ventral ACC	−145.11	−145.44	.9192
Caudate–subcallosal ACC	−170.53	−170.95	.8908
Caudate–frontal medial	−166.66	−166.38	.3231
Caudate–accumbens	−149.76	−150.91	.8908
Putamen–dorsal ACC	−138.38	−138.16	.8617
Putamen–ventral ACC	−190.79	−190.22	.7201
Putamen–subcallosal ACC	−165.07	−165.90	.8908
Putamen–frontal medial	−188.42	−187.88	.0469^*^
Putamen–accumbens	−201.99	−201.95	.8908
Putamen–caudate	−117.36	−117.32	.8908
Amygdala–dorsal ACC	−111.87	−111.07	.2559
Amygdala–ventral ACC	−98.59	−96.12	.3231
Amygdala–subcallosal ACC	−9.05	−9.11	.9838
Amygdala–frontal medial	−172.22	−171.99	.5354
Amygdala–accumbens	−117.48	−117.58	.8908
Amygdala–caudate	−134.94	−134.83	.7360
Amygdala–putamen	−122.05	−123.67	.4784
Hippocampus–dorsal ACC	−147.86	−144.27	.0092^*^
Hippocampus–ventral ACC	−87.71	−88.02	.8908
Hippocampus–subcallosal ACC	−25.88	−33.14	.2559
Hippocampus–frontal medial	−81.77	−81.69	.7201
Hippocampus–accumbens	−148.25	−158.80	.1217
Hippocampus–caudate	−211.57	−213.21	.3516
Hippocampus–putamen	−184.59	−185.58	.8908
Hippocampus–amygdala	−73.36	−71.89	.4614

Abbreviations: ACC = anterior cingulate cortex; BIC = Bayesian Information Criterion; PDS = Pubertal Development Scale.

*Note*. Multiple comparison corrected *p*‐values are reported (Bonferroni–Holm). *Corrected *p*‐value <.05.

## 4 DISCUSSION

This study examined longitudinal changes within and between subcortical–cortical connectivity across adolescent development extending into young adulthood (8–29 years). Given that prior studies testing subcortical reactivity suggested that puberty may be a driving factor for nonlinear age patterns (Crone & Dahl, 2012; Ladouceur, 2012; Pfeifer et al., 2011), an important question was whether development would be better described by pubertal development than age. For this purpose, we made use of a three‐wave accelerated longitudinal data set with boys and girls across different stages of pubertal development, who completed RS scans at each time point.

We observed several key findings. First, a quarter of the subcortical–cortical connections we investigated weakened with age. The decrease in subcortical–cortical connectivity was unique, given that functional connectivity between subcortical and between cortical regions only strengthened with age. Second, for several key connections the decrease in subcortical–cortical connectivity was better described by pubertal development than age, suggesting that puberty may be one of the mechanisms that initiate change in subcortical–cortical development. The discussion is organized alongside these main findings.

### 4.1 Age‐related changes in functional connectivity

To examine functional connectivity patterns in adolescence, we specified regions as part of subcortical and medial cortical reward regions typically implicated in functional reward processing and motivated behavior (Doremus‐Fitzwater & Spear, 2016; Haber & Knutson, 2010; Telzer, 2016; van Duijvenvoorde, Achterberg, et al., 2016). Our results showed, first, that connectivity patterns were highly stable across time points at the group level, with excellent within‐sample replication of positive and negative connectivity patterns across three time points. Second, the within‐individual stability was relatively low, with ICCs ranging between 0.08 and 0.33, although most being at least above .1 (e.g., Ordaz, Foran, Velanova, & Luna, 2013). Compared to fMRI ICCs, these values are comparable for neural activity in subcortical brain regions (Braams et al., 2015; Herting, Gautam, Chen, Mezher, & Vetter, 2017; Schreuders et al., 2018) and highlight that patterns were more consistent at the group than the individual level. One interpretation of low test–retest reliability (such as the ICC) is that there is a poor consistency of functional connectivity. However, for studies with relatively longer delays between time points and younger populations, a low ICC may also reflect development over time (Herting et al., 2017). To distinguish which of these effects contributes most to these ICC results, future studies should further examine the test–retest reliability of RS scans in developmental populations, preferably also including shorter durations between scans.

A main goal in this study was whether RS functional connectivity changed over the course of adolescent development. Results showed that the patterns of change were dependent on the specific connection that was studied. More specifically, we observed an age‐related decline in functional connectivity between the nucleus accumbens, the putamen, and more ventral regions of the medial PFC, (i.e., subcallosal medial cortex and frontal medial cortex). These findings fit well with prior cross‐sectional reports (Fareri et al., 2015; van Duijvenvoorde, Achterberg, et al., 2016), and have been interpreted as a more independent functioning of these networks involved in affective‐motivational processes. Decreases in functional connectivity with age were also observed for structures such as the caudate with the dorsal ACC, which concurs with a more dorsal‐to‐ventral divide of striatal connectivity (Di Martino et al., 2008; Porter et al., 2015). These patterns were paralleled by increases in connectivity between subcortical regions and between cortical regions. These findings fit prior research that has shown strengthening of functional connectivity in cortico‐cortical connections (Supekar, Musen, & Menon, 2009), as well as subcortico‐subcortical connectivity (van Duijvenvoorde, Achterberg, et al., 2016), and extend previous findings by providing longitudinal evidence for stronger integration within subcortical and within cortical regions across development. Although GAMM is a descriptive test of age effects, inspection of the best‐fitting age curves (Figure 4) from our models indicates that for specific connections developmental changes in connectivity follow a nonlinear pattern, with changes leveling off in early adulthood. This “leveling off” was particularly pronounced for decreases in connectivity between the putamen–frontal medial cortex, caudate–dorsal ACC, and hippocampus–dorsal ACC. This may suggest most prominent changes in adolescence within this decoupling of these functional networks.

Together, these findings may be interpreted as change in local versus distributed networks in which specific functional networks both strengthen and weaken across adolescence. Previous work also showed that structural networks become more segregated across development (Baum, Ciric, Roalf, Betzel, et al., 2017), and that BOLD dimensionality decreases with age (Kundu et al., 2018). One suggested interpretation is that these changes may maximize the neural efficiency of interregional communication (Stevens, 2016). Future studies may complement our RS functional connectivity findings by using more explorative analyses such as graph‐theory models together with age‐ and pubertal development, allowing examination of metrics of brain organization and neural efficiency on a whole‐brain basis.

### 4.2 Age‐ versus puberty‐related changes in functional connectivity

Previous studies focusing on functional reactivity have often suggested that pubertal development may advance or enhance growth trajectories of brain development, although most studies up to now focused on brain structural development (e.g., Herting & Sowell, 2017; Vijayakumar, Mills, Alexander‐Bloch, Tamnes, & Whittle, 2018) or cross‐sectional functional connectivity development (Fareri et al., 2015). Here, we observed that a number of subcortical–cortical connections sensitive to developmental change were better described by (self‐reported) pubertal development than age. That is, in boys we observed that the decrease in connectivity between the nucleus accumbens with the frontal medial cortex was better described by pubertal development than age. Girls showed a similar effect of pubertal development on putamen and medial PFC connectivity, and, additionally, on connectivity between the caudate and dorsal ACC. Finally, a few specific connections were explained by pubertal development over and above baseline age. For boys, this was only the strengthening between hippocampus–amygdala connectivity and for girls the decoupling between hippocampal–dorsal ACC and caudate–dorsal ACC connectivity.

Until now, a large body of work on pubertal effects on neural development is based on animal studies. Recent animal evidence suggests that puberty may be a critical driver in reward‐circuitry development. For instance, animal work has observed a reduction in medial PFC volume and synapses in postpubertal rats, and neuronal losses during pubertal onset (Walker et al., 2017; Willing & Juraska, 2015). In humans, decreases in gray matter density in frontal regions (Peper et al., 2009), as well as hippocampus, amygdala, and caudate volumes (Goddings et al., 2014; Wierenga et al., 2018) have been related to pubertal development, but—to our knowledge—these findings are one of the first to test and compare effects of pubertal development on RS functional connectivity (but see also Ernst et al., 2019). Including pubertal development improved model fits for changes in specific subcortical–cortical connections, suggesting that the developing efficiency of the brain is a puberty‐driven maturational processes that may accelerate changes in modularity and plasticity in the developing brain. These differences between sexes may suggest that pubertal development in boys and girls has differential influence on the development of subcortical–cortical connectivity. A recent study into RS connectivity in the cortical default network observed particularly sex × pubertal developmental interactions in which connectivity decreased across pubertal development in girls, whereas it increased in boys (Ernst et al., 2019). This was tentatively interpreted as relevant to the emergence of affective dysregulation in adolescence that affect girls more. Although we did not explicitly test for sex × pubertal developmental interactions, our findings seem to indicate that pubertal development decreases connectivity in both girls and boys, yet affects different subcortical–cortical connections. When controlling for baseline age, however, pubertal development in boys particularly strengthened amygdala–hippocampal connectivity, and for girls particularly decreased connectivity with the dorsal ACC. Future studies will need to extend and replicate these findings in male and female pubertal cohorts that have been followed on an individual level from prepubertal to postpubertal development.

Note that when testing sex differences, we observed only few differences in functional connectivity between boys and girls. That is, amygdala–hippocampus connectivity, a connection most prominently influenced by sex, was stronger for boys than for girls, and hippocampal–ventral ACC connectivity was stronger for girls than for boys. Previous research on sex differences has mostly been done in adults (e.g., Alarcón, Cservenka, Rudolph, Fair, & Nagel, 2015; Kogler et al., 2016), and has shown higher hippocampal and/or amygdala connectivity in females than males, possibly related to their better memory performance (Gur & Gur, 2016). The sex differences we observed here may be an interesting starting point, but should be interpreted with caution until replicated, given the lack of consistent findings in prior developmental samples.

### 4.3 Limitations

RS connectivity is inherently susceptible to effects of motion, which can have a marked influence on developmental findings. In the current study, we have taken steps to account for such possible confounds (see Satterthwaite et al., 2017). Specifically, we first excluded people above our set motion threshold. Further, we included realignment parameters, tissue‐specific signals, global signal regression, and a denoising procedure based on an independent components analyses (FSL FIX). One possible concern is that this latter denoising procedure has mainly been applied in adult populations. However, the use of these denoising techniques may be especially helpful in cleaning relatively noisy data from such specific populations, and can improve signal and analysis quality. Applying control analyses on motion confounds in our cleaned time series indicated that FD was minimalized across all time points, supporting its use in the current data set. Another concern is the inclusion of global‐signal regression. Regressing out global signal may reduce noise from physiological measures such as heart rate and respiration (Chen et al., 2012; Power et al., 2015) and differences in vigilance and arousal (Liu et al., 2017), yet it has been found to induce negative correlations and spurious results (Satterthwaite et al., 2013). Explicit comparisons in another developmental RS study, showed very high comparability (*r* = .94; Gabard‐Durnam et al., 2014) when comparing results with or without global signal regression. Thus, to benefit from the reduction in artifacts, as well as to build on prior developmental studies (Fareri et al., 2015; Gabard‐Durnam et al., 2014; Peters et al., 2015; Peters, Peper, et al., 2016; Peters, van Duijvenvoorde, Koolschijn, & Crone, 2016; van Duijvenvoorde, Achterberg, et al., 2016), we opted to also include a global signal regression.

A second limitation of our analyses may be that we used a set of anatomical ROIs based on the Harvard–Oxford atlas. Using anatomical ROIs versus a functional brain atlas (such as the areal atlas of Power et al., 2011) may be less sensitive in detecting age‐ (or puberty) related change. Parcellation studies in adults have identified that structural atlases suffer from lower homogeneity than functional parcellations (Craddock, James, Holtzheimer, Hu, & Mayberg, 2012; Gordon et al., 2014). This is particularly so for atlases that use large structural regions such as the AAL (Gordon et al., 2014), while more fine‐grained structural atlases such as Brodmann areas seem to perform better. The advantage of structural atlases is that they are highly standardized, and used typically in both developmental functional imaging studies (e.g., Achterberg et al., 2018) and RS studies (e.g., Fareri et al., 2015; Stevens, 2016). Here, we used an anatomical atlas that may allow for more specific cortical regions by choosing a standardized structural probabilistic atlas, namely the Harvard–Oxford atlas. Nonetheless, future studies should consider comparing these functional and structural approaches in a developmental perspective.

## 5 CONCLUSION

The current study used RS functional connectivity in a large longitudinal sample to understand developmental changes in connectivity between and within subcortical and medial prefrontal regions. These findings have implications for future research: they confirm patterns of subcortical–cortical connectivity changes, and advance insights by suggesting an important role for pubertal development in the development of subcortical–cortical functional connectivity. This may be an important starting point for further understanding of hormonal effects operating on functional connectivity development, and the link with real‐life reward‐driven behaviors.

## CONFLICT OF INTEREST

The authors declare no conflict of interest.

## Data Availability

The data that support the findings of this study are available from the corresponding author upon reasonable request.
